# Cushing's Syndrome in Pregnancy Secondary to Adrenocortical Adenoma: A Case Series and Review

**DOI:** 10.1002/edm2.474

**Published:** 2024-03-12

**Authors:** Yan Wang, Yufen An, Xiaomei Hou, Yanan Xu, Zhen Li, Xin Liu, Fumin Zheng, Mingze Sun, Rendong Han, Caixia Lu, Jing Li, Jun Zhou

**Affiliations:** ^1^ Department of Obstetrics The Affiliated Hospital of Qingdao University Qingdao China; ^2^ Department of Pathology The Affiliated Hospital of Qingdao University Qingdao China

**Keywords:** adrenocortical adenoma, Cushing's syndrome, pregnancy

## Abstract

**Purpose:**

To present a case series of Cushing's syndrome (CS) during pregnancy caused by adrenocortical adenomas, highlighting clinical features, hormonal assessments and outcomes.

**Methods:**

We describe five pregnant women with CS, detailing clinical presentations and laboratory findings.

**Results:**

Common clinical features included a full moon face, buffalo back and severe hypertension. Elevated blood cortisol levels with circadian rhythm disruption and suppressed adrenocorticotrophic hormone (ACTH) levels were observed. Imaging revealed unilateral adrenal tumours. Two cases underwent laparoscopic adrenalectomies during the second trimester, while three had postpartum surgery. All required hormone replacement therapy, with postoperative pathological confirmation of adrenocortical adenomas.

**Conclusion:**

Diagnosis of CS during pregnancy is challenging due to overlapping features with normal pregnancy: elevated blood cortisol levels and abnormal diurnal rhythm of blood cortisol, suppressed aid diagnosis. Treatment should be individualised due to a lack of explicit optimum therapeutic approaches. Laparoscopic adrenalectomy may be an optimal choice, along with multidisciplinary management including hormone replacement therapy.

## Graphical abstract

A case series of five pregnant women diagnosed with Cushing's syndrome (CS) caused by adrenocortical adenoma, highlighting clinical features, hormonal assessments and outcomes was presented. This review explored the clinical features, pathogenesis, treatment approaches and prognosis of CS during pregnancy caused by adrenocortical adenoma.

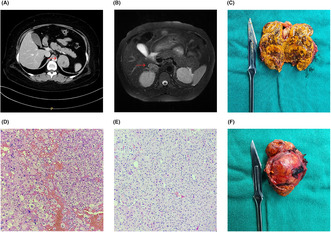

## 1 Introduction

Endogenous Cushing's syndrome (CS) during pregnancy is an infrequent condition characterised by excessive secretion of cortisol hormones, which can be detrimental to pregnant women and their developing foetuses. If not promptly diagnosed and adequately treated, it can lead to high maternal and foetal morbidity and mortality [1, 2]. The primary cause of CS during pregnancy is adrenal adenoma, with pituitary aetiology and adrenal carcinoma being uncommon causes [3]. Excessive secretion of cortisol hormones disrupts the normal functioning of the hypothalamic‐pituitary‐adrenal (HPA) axis and leads to hypogonadotropic hypogonadism, which is characterised by impaired ovulation, menstrual irregularities and even amenorrhea [4, 5]. Common signs of CS during pregnancy include a buffalo hump, a full moon face and striae in the abdomen and legs. Sometimes, high blood pressure is the primary manifestation. However, these signs can also be seen in a healthy pregnancy, and identifying and diagnosing CS during pregnancy is complicated due to nonspecific signs and the lack of timely screening, especially when diabetes or hypertension is also present. However, screening for CS in diabetes or hypertension women is not recommended unless there is a clinical suspicion for CS. The effective management of this condition requires a multidisciplinary team consisting of maternal‐foetal medicine specialists, endocrinologists and surgeons [6]. Laparoscopic adrenalectomy may be the preferred treatment for CS except in cases of contraindications [7]. Here, we present five cases of CS during pregnancy caused by adrenocortical adenomas at our institution, review the literature, illustrate the pathogenesis and provide recommendations on detection and management.

## 2 Case Review

Data from five cases of pregnancy combined with adrenocortical adenomas admitted to the Affiliated Hospital of Qingdao University from August 2016 to June 2023 were selected for analysis. A retrospective approach was employed to gather patients' general information, clinical characteristics, treatment protocols and maternal and infant outcomes (Tables 1 and 2). Due to a retrospective study, patients were exempted from giving informed consent.

**TABLE 1:** General information in five pregnant patients with Cushing's syndrome.

Case	Age (years)	Time of presentation (weeks)	Blood pressure (mmHg)	Obstetric score	BMI	Glucose	Time of termination (weeks)	Method of termination	Time of laparoscopic (weeks)	Foetal outcome (Apgar scores)
1	24	23	165/93	G1P0	31.25	DM	37	Caesarean	25 + 4	10‐10‐10
2	39	22 + 5	180/111	G1P0	32.04	GDM	34 + 5	Caesarean	24 + 3	10‐10‐10
3	35	17	167/105	G2P1	40.56	DM	17 + 3	Induce labour	Postpartum	Foetal demise
4	30	36 + 2	158/118	G1P0	33.20	Normal	36 + 2	Caesarean	Postpartum	10‐10‐10
5	27	29 + 3	179/114	G1P0	28.40	GDM	31 + 3	Caesarean	Postpartum	10‐10‐10

**TABLE 2:** Results of biochemical screening and clinical characteristics of five patients with Cushing's syndrome.

Case	Time of evaluation (weeks)	Diurnal cortisol rhythm (nmol/L) (8 am‐4 pm‐12 am) (normal range: 166–507)	ACTH (pg/mL) (8 am‐4 pm‐12 am) (normal range: 7.2–63.3)	Aldosterone (pg/mL) (normal range: 30–160)	Renin activity (ng/mL/h) (normal range: 0.15–2.33)	1 mg‐DST (nmol/L)	Urinary protein	Pituitary magnetic resonance	Adrenal magnetic resonance/computerised tomography
1	23 + 2	543.00‐594.00‐553.00	2.10‐2.97‐1.89	67.49	1.99	604.00	Negative	Negative	Left
									21.9 × 25.0 mm
2	23	701.00‐731.00‐713.00	1.50‐1.50‐1.50	20.96	6.53	698.00	Negative	Negative	Right
									24.0 × 21.0 mm
3	17 + 1	650.00‐649.00‐680.00	1.52‐1.60‐1.59	184.50	15.44	589.00	Positive	Negative	Left
									50.0 × 33.0 mm
4	Postpartum	617.59‐659.31‐611.8	5.02‐1.14‐1.61	52.7	0.18	—	Negative	Negative	Left
									38.0 × 52.0 mm
5	29 + 5	1239.00‐1190.00‐1176.00	<1.0‐<1.0‐<1.0	—	—	—	Negative	Negative	Right
									32.0 × 27.0 × 32.0 mm

### 2.1 Case 1

Patient 1, a 24‐year‐old primigravida, was admitted to the endocrinology department at the 23rd week of gestation due to the presence of distensible striae on the skin and a noticeable increase in weight. Upon examination, she had a considerable number of purple striae on her abdomen and thighs as well as a full moon face and buffalo hump. Her BMI was calculated to be 31.25 kg/m^2^. On admission, her blood pressure was measured at 165/93 mmHg, and her fasting blood glucose level was 8.0 mmol/L, requiring insulin for control. She had fluctuating blood potassium levels, ranging from 2.7 to 3.2 mmol/L (normal range: 3.5–5.5 mmol/L), and urinalysis showed no signs of proteinuria. Her treatment included administering 100 mg of labetalol three times daily to control blood pressure and intravenous potassium supplementation to correct hypokalaemia (Table 1).

**FIGURE 1:**
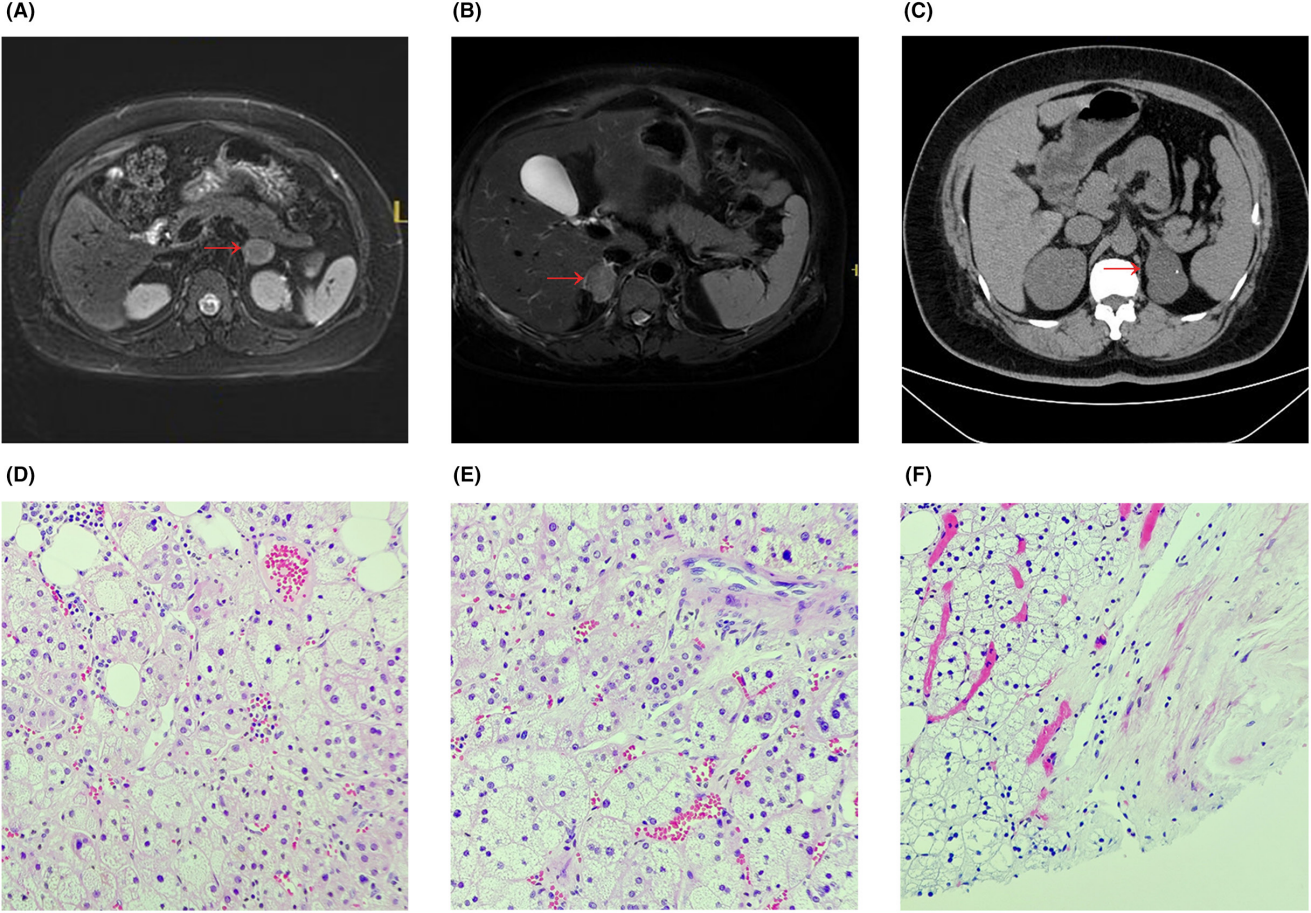
MRI scans (A, B) and CT scans (C) reveal the presence of an adrenal tumour, indicating the possibility of adrenal adenoma in Patient 1 (A), Patient 2 (B) and Patient 3 (C). A haematoxylin and eosin staining (H&E) of adrenocortical adenomas in pregnant patients (magnification 40×). (D) Adrenal adenoma from Patient 1. (E) Adrenal adenoma from Patient 2. (F) Adrenal adenoma from Patient 3.

Endocrine indicators were measured at 8:00, 16:00 and 00:00 hours. The results showed plasma cortisol levels of 543.00, 594.00 and 553.00 nmol/L (normal range: 166–507 nmol/L). ACTH levels during the same period were 2.10, 2.97 and 1.89 pg/mL (normal range: 7.2–63.3 pg/mL). Aldosterone levels were detected at 67.49 pg/mL (normal range: 30–160 pg/mL), and renin activity was measured at 1.99 ng/mL/h (normal range: 0.15–2.33 ng/mL/h). A 1‐mg dexamethasone suppression test uncovered that the plasma cortisol levels were not suppressed (Table 2). The result of an MR scan of the pituitary did not indicate any abnormalities. However, an MR scan of the adrenal gland without contrast revealed a left adrenal tumour measuring approximately 21.9 × 25.0 mm (Figure 1).

After a comprehensive consultation involving the endocrinology, urology and obstetrics departments, she was diagnosed with ACTH‐independent CS, indicating an adrenocortical adenoma. As a result, she underwent laparoscopic adrenalectomy at the 25th gestational week and 4 days. The postoperative pathology confirmed the presence of an adrenocortical adenoma (Figure 1). Following the surgery, she was prescribed hydrocortisone and labetalol. At the 37th week of gestation, a caesarean section was successfully performed due to uncontrolled hypertension. The neonatal Apgar scores were recorded as 10 at both the first and fifth minutes after birth. After the surgery, she experienced a smooth recuperation and was discharged on the fourth day.

At the 42nd day after delivery, the patient exhibited stable and well‐controlled blood pressure and blood glucose levels, which allowed for the discontinuation of her antihypertensive medication. Additionally, her serum potassium levels returned to normal, and the dosage of hydrocortisone was gradually reduced. Further assessment of her hormonal levels at the sixth month postpartum revealed normal ACTH levels, displaying a successful resolution of the previous adrenal tumour.

### 2.2 Case 2

Patient 2, a 39‐year‐old woman who had conceived through embryo transfer for infertility, was admitted to the hospital due to uncontrolled hypertension at the 22nd week and 5 days of gestation. A foetal systemic ultrasound at the 24th week of gestation revealed placenta previa. Additionally, at 25th week of gestation, an oral glucose tolerance test (OGTT) confirmed gestational diabetes mellitus.

The clinical picture was supported by the presence of abdominal obesity, fat pads on the back and purple striae on the abdomen. Her BMI was calculated to be 32.04 kg/m^2^. In addition, her blood pressure reading of 180/111 mmHg was a cause for concern. Upon admission, the patient's urinalysis showed an absent urine protein, while liver and renal function, as well as platelet count, were within normal ranges, ruling out the presence of HELLP syndrome. To manage her blood pressure, urapidil was administered intravenously. The repeated occurrences of hypokalaemia raised concerns, leading to an endocrine evaluation. Aldosterone and renin levels were within normal ranges, whereas plasma ACTH levels were below the normal range. The plasma cortisol rhythm showed abnormalities, and both low‐ and high‐dose dexamethasone tests did not suppress plasma cortisol secretion (Tables 1 and 2). Further investigations were conducted, including an MRI scan that showed no craniocerebral abnormalities but revealed a right adrenal tumour measuring approximately 24.0 × 21.0 mm, indicating ACTH‐independent CS (Figure 1).

At her 24th week of gestation, she underwent a laparoscopic adrenalectomy, which revealed the presence of an adrenocortical adenoma based on subsequent pathology results (Figure 1). Following the procedure, hydrocortisone replacement therapy was initiated. At the 34th week and 5 days of pregnancy, a caesarean section was performed due to vaginal bleeding caused by placenta previa. Despite neonatal Apgar scores of 10 at both the first and fifth minutes after birth, the neonate was admitted to the paediatric unit due to prematurity. After 4 days, she was discharged while her antihypertensive medication was gradually reduced. However, she is still undergoing hormone replacement therapy and is currently being observed during follow‐up.

### 2.3 Case 3

At the 17th week of gestation, a 35‐year‐old multipara presented to the emergency department with severe shortness of breath after physical activity. Further investigation using cardiac ultrasound revealed impaired left heart function, with an ejection fraction of 48%. Upon admission, her BMI was 40.56 kg/m^2^. Her blood pressure was measured at 167/105 mmHg, and her urinary protein level was positive. Unfortunately, she was receiving an irregular dosage of labetalol, 50 mg administered every 12 h. Additionally, for 12 years she had a history of diabetes mellitus and was being treated with insulin. Taking into account her full moon face, hypokalaemia and difficulties in controlling her blood pressure, an evaluation for CS was recommended. The results of the test indicated a blunt plasma cortisol rhythm. Further examination through ultrasound revealed the presence of a mass in the left adrenal gland, which raised the possibility of an adrenal tumour (Tables 1 and 2).

Due to reduced heart function and uncontrolled hypertension, she was terminated the pregnancy at 17th week and 3 days of gestation. Approximately 1 month later, a left adrenal tumour measuring approximately 50 × 33 mm was discovered through an adrenal computed tomography (CT) scan. As a result, she underwent a laparoscopic adrenalectomy. The postoperative pathology report confirmed that the tumour was a cortical adenoma (Figure 1). To avoid adrenal insufficiency following adrenalectomy, oral hydrocortisone was administered after the surgery. Currently, the patient is being regularly monitored to ensure optimal postoperative care and track her progress.

### 2.4 Case 4

Patient 4, a 30‐year‐old unipara, was admitted to the hospital for premature rupture of membranes at the 36th week and 2 days of gestation. She had a history of systemic lupus erythematosus (SLE) for the preceding 4 years. During pregnancy, she was prescribed 12 mg/day methylprednisolone, 0.4 mg/day hydroxychloroquine sulphate and 1.5 mg/day tacrolimus. Upon admission, her BMI was 33.20 kg/m^2^, with an abdominal obese body type and a rounded face; her blood pressure was measured at 158/118 mmHg, and urinary protein was negative. Moreover, she had an obvious hypokalaemia (Tables 1 and 2). Subsequent examination using urinary ultrasound revealed a mass in the left adrenal, raising suspicion of an adrenal tumour. She underwent a successful caesarean section on the second day, was discharged from the hospital and discontinued methylprednisolone on the third day after the operation.

**FIGURE 2:**
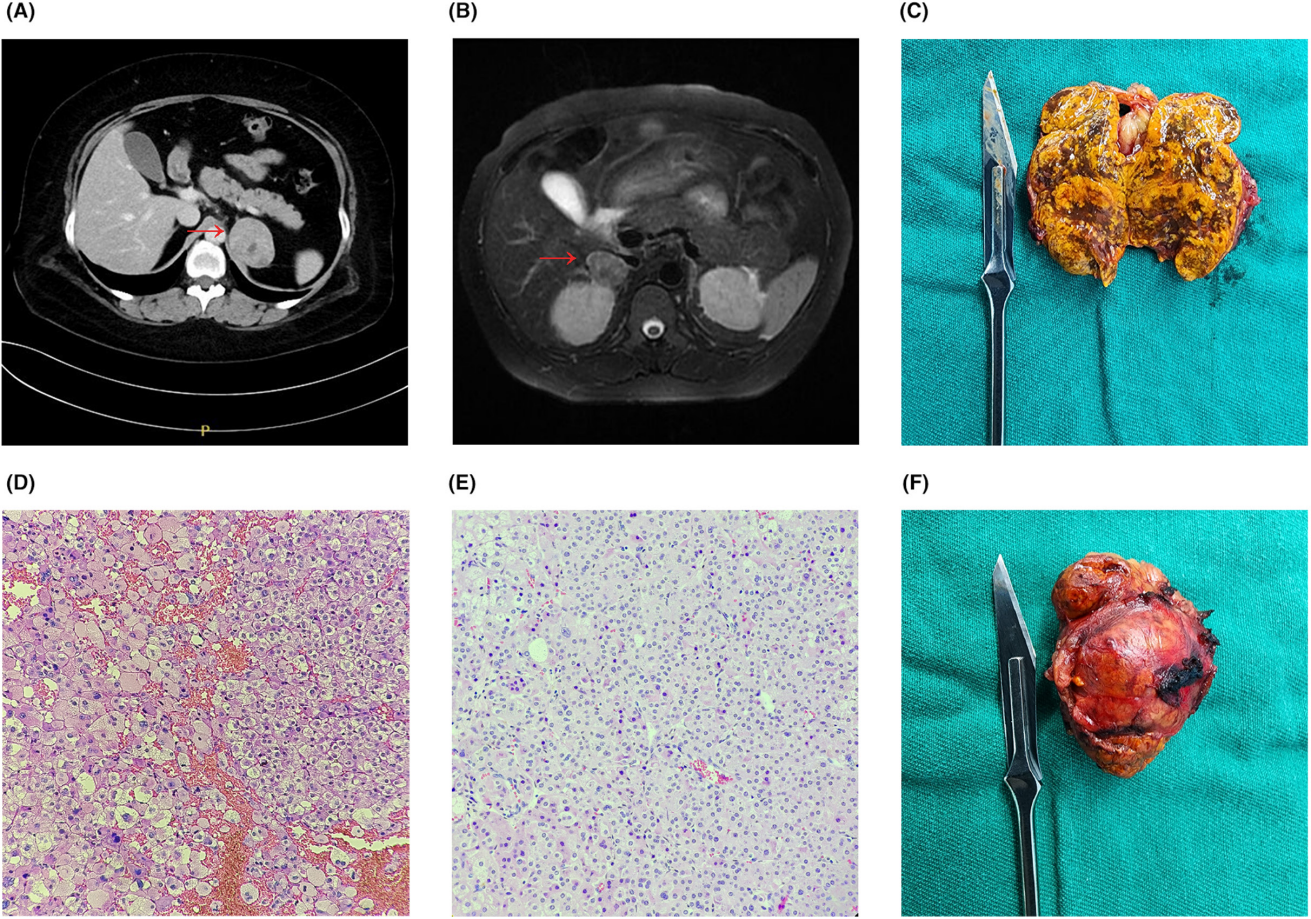
CT scans (A) and MRI scans (B) reveal the presence of an adrenal tumour, indicating the possibility of adrenal adenoma in Patient 4 (A) and Patient 5 (B). A haematoxylin and eosin staining (H&E) of adrenocortical adenomas in pregnant patients (magnification 40×). (D) Adrenal adenoma from Patient 4. (E) Adrenal adenoma from Patient 5. (C) and (F) are intraoperative views of adrenal adenoma during the dissection of Patient 5.

One year later, she presented to the urology department with uncontrolled blood pressure. Additionally, she experienced occasional symptoms of palpitations and chest stuffiness. The endocrine findings suggested ACTH‐independent hypercortisolism (Table 2). A CT evaluation revealed a 38.0 × 52.0 mm tumour on the left adrenal gland. She underwent laparoscopic excision of the left adrenal gland, and the specimen was confirmed to be a cortical adenoma through postoperative pathology (Figure 2). Following the surgery, she was prescribed hydrocortisone, but it was discontinued after 1 year. Subsequent evaluations showed that her cortisol and ACTH levels had returned to normal.

### 2.5 Case 5

Patient 5, a 27‐year‐old primipara, presented to the obstetric department at the 29th week and 3 days of gestation due to substantially elevated blood pressure and bilateral lower limb oedema that had developed 1 month prior. The OGTT showed glucose levels of 4.33‐12.49‐6.1 mmol/L, which were effectively managed through dietary control. On examination, she exhibited a markedly elevated blood pressure of 179/114 mmHg, negative urinary protein, an obese body type with the BMI of 28.4 kg/m^2^, central distribution of adipose tissue, a rounded and full moon face, acne and visible purple lines on the abdomen (Table 1). Upon evaluation, her potassium ion levels were found to be 2.7 mmol/L (normal range: 3.5–5.5 mmol/L). To manage her condition, she received sedation, antihypertensive and antispasmodic medication, and potassium supplementation. Further testing revealed a substantial increase in cortisol concentration and the absence of the circadian rhythm, while ACTH levels were markedly suppressed (Table 2). Imaging results showed the presence of a right adrenal adenoma measuring approximately 32.0 × 27.0 × 32.0 mm (Figure 2). Due to her unmanageable blood pressure, a caesarean section was performed at the 31st week and 3 days of gestation.

She underwent laparoscopic adrenalectomy 42 days after pregnancy termination. Postoperative pathology confirmed the presence of a cortical adenoma (Figure 2). Hormone replacement therapy was administered, and electrolytes and cortisol levels were monitored on postoperative Days 1, 2 and 5. It was observed that these levels returned to normal. Subsequent follow‐up visits confirmed stable blood glucose and blood pressure.

## 3 Discussion

CS during pregnancy is a rare condition, with only 250 reported cases documented in the literature [8]. The physiological changes that occur in pregnant women make it more likely for this condition to be ignored or misdiagnosed, especially the existence of diabetes or hypertension, which can have negative effects on both the pregnant woman and the developing foetus. Therefore, early diagnosis and effective treatment are crucial in mitigating potential harm.

### 3.1 Aetiology

Endogenous CS is typically divided into two types based on its cause: ACTH‐dependent CS and ACTH‐independent CS. The majority of ACTH‐dependent CS cases are caused by pituitary adenomas that produce ACTH, known as Cushing's disease, and this accounts for approximately 75% of all CS cases. Ectopic ACTH secretion is rare. It is worth noting that Cushing's disease is more commonly observed in nonpregnant individuals than in pregnant patients [9]. Active CS produces large amounts of cortisol hormones that impact the reproductive system by affecting the female HPA axis to decrease circulating levels of LH [10]. ACTH‐dependent CS is often associated with hyperandrogenism. ACTH‐dependent CS product excessive levels of androgens hinder the production of gonadotrophins, which disrupt normal follicular development, resulting in anovulation, amenorrhea and, ultimately, infertility. In contrast, ACTH‐independent CS generally generates cortisol alone, lightly impacting ovulatory function [11]. ACTH‐independent CS primarily occurs due to adrenocortical adenomas, which make up approximately 15% of CS cases (compared with 40%–60% in pregnant individuals) [5, 12]. Bilateral adrenal hyperplasia and adrenal adenocarcinoma are scarce causes [9].

### 3.2 Pathophysiology

Studies have shown that cortisol secretion by adrenal adenomas is regulated by genetic or molecular mechanisms. One of the key factors in this regulation is the aberrant expression of G protein–coupled hormone receptors (GPCR) in the adrenal cortex. These receptors, such as vasopressin, catecholamine, luteinising hormone/human chorionic gonadotropin (LHCG) and serotonin receptors, are functionally coupled to steroidogenesis and play a significant role in cortisol secretion [13]. In addition, other receptors like angiotensin, leptin, glucagon, IL‐1 and TSH receptors have also been identified in this process [14]. Oestrogen receptor α (ERα) also plays an important role in CS in pregnancy. In research studies, scholars have demonstrated the presence of ERα in the adrenal cortex and adenomas and have observed that oestrogen levels increase the expression of these receptors in adrenal adenomas during pregnancy. This upregulation of oestrogen receptor expression in adrenal adenomas has the potential to enhance cortisol secretion by adrenocortical cells and contribute to the proliferation of adrenocortical cells [15]. The high expression of Ki‐67 in adrenocortical adenomas is a reliable marker of proliferation. Pregnancy‐induced CS overexpressing LHCGR, elevated HCG levels during pregnancy stimulate aberrant LHCGR expression in adrenal adenoma tissue, leading to the activation of the cyclic adenosine monophosphate (cAMP) and protein kinase A (PKA) pathways. This activation enhances cortisol production in adrenal cortical cells [16]. Studies have indicated that genetic alterations in the catalytic subunit of PKA are linked to various human diseases. Mutations in *PRKACA*, the gene responsible for encoding the primary catalytic subunit of PKA, result in the activation of PKA in somatic cells. When somatic *PRKACA* is duplicated in the germline, it leads to the development of bilateral adrenal adenomas. On the other hand, mutations in this gene give rise to unilateral cortisol‐producing adrenal adenomas [17]. At the same time, it is possible that altered DNA methylation and insulin‐like growth factor 2 (IGF2) overexpression are involved in the progression of adrenocortical tumours and the promotion of cell proliferation [18, 19].

### 3.3 Clinical Features

There is a substantial overlap between CS and normal pregnancy symptoms, such as weight gain, centripetal obesity and skin striae. As a result, it is possible to miss the identification of CS in pregnant patients [8]. Hypertension and hyperglycaemia are common in both normal pregnancy and CS, and their prevalence in CS has been reported to be quite substantial, with rates as high as 68% and 25.0%, respectively [20]. These alarming statistics highlight the important impact that CS can have on the safety of both the pregnant woman and the developing foetus [12, 21]. In some cases, patients may present with uncontrollable hypertension as their primary symptom, including gestational hypertension and preeclampsia. To effectively manage and control hypertension in each patient, targeted therapeutic interventions were implemented. CS and preeclampsia share the common feature of hypertension; preeclampsia is typically the first consideration for clinicians when evaluating patients with this symptom despite the presence of hypokalaemia. Table 3 provides a comparison of clinical characteristics between CS and preeclampsia during pregnancy. In addition to the typical external features, CS is often associated with hypokalaemia and hyperglycaemia. However, oedema and proteinuria are common symptoms in hypertensive pregnant patients, including abnormal liver function and coagulation. In a small subset of cases, CS can lead to complications such as poor wound healing, osteoporosis, fractures and severe psychiatric disorders. Untreated CS substantially increases the risk of maternal mortality, with estimates suggesting a staggering 50% mortality rate within 5 years [4, 5]. Furthermore, there is a notable rise in foetal complications linked to CS. They encompass low weight at birth, intrauterine growth retardation (IUGR), instances of spontaneous abortion, preterm birth and a perinatal neonatal mortality rate of 8.8% [4]. It is worth mentioning that the actual incidence of adverse outcomes might be even higher due to difficulties in promptly diagnosing CS during pregnancy.

**TABLE 3:** Differentiating features between preeclampsia and CS.

Feature^a^	CS	Preeclampsia
*Signs and symptoms*		
Time of presentation	Anytime during pregnancy	>20 weeks of gestation
Hypertension	Usually, uncontrolled	Usually, controlled
Bipedal oedema	Absent	May be present
Buffalo hump	Present	Absent
Full moon face	Present	Absent
Red‐purple skin striae	Present	Absent
Weight gain	Present	Absent
*Laboratory finds*		
Proteinuria	May be present	Present
Glucose	Elevated	Normal
Plasma potassium	Depressed	Normal
Liver transaminases	Normal	Elevated
Plasma cortisol	Elevated and rhythm disappeared	Normal
ACTH	Depressed	Normal

^a^ Data showing common clinical and laboratory features to distinguish disease states.

### 3.4 Diagnosis

When diagnosing CS, screening measures are necessary if typical clinical features are present in a patient. Meanwhile, the presence of both uncontrollable hypokalaemia and hypertension could serve as a warning sign for CS. In nonpregnant women, the focus is on identifying elevated cortisol levels, loss of the normal circadian rhythm and the inability to effectively suppress cortisol production with low doses of dexamethasone [12]. However, during pregnancy, the screening for CS becomes difficult due to the active HPA axis leading to a rise in corticotropin‐releasing hormone (CRH), ACTH, and free and total cortisol levels [22]. As placental oestrogen levels rise, there is an increase in the production of hepatic corticosteroid‐binding globulin (CBG), which in turn stimulates cortisol production. Compared with prepregnancy, levels of circulating and urinary‐free cortisol (UFC) concentrations obviously increase throughout pregnancy, with a two‐ to threefold in the middle and third trimesters. Therefore, if the 24‐h UFC levels exceed 3 times the upper reference range, CS should be strongly suspected [23]. During pregnancy, the increasing levels of CBG and accompanying cortisol reduce the inhibition of cortisol release in response to dexamethasone, which may lead to false‐positive results. According to the guidelines of the Endocrine Society, the use of the overnight or low‐dose dexamethasone suppression test (DST) as an initial assessment for CS in pregnant women is not recommended [24]. Despite the changes in the HPA axis, the circadian rhythm of cortisol secretion remains. However, patients with CS experience a loss of normal diurnal variation, which can be diagnosed by measuring late‐night salivary cortisol (LNSC) [25]. Lopes LML established upper reference limits for LNSC during pregnancy, which are 6.9, 7.2 and 9.1 nmol/L for the first, second and third trimesters, respectively [26]. However, further experimental data are needed.

**FIGURE 3:**
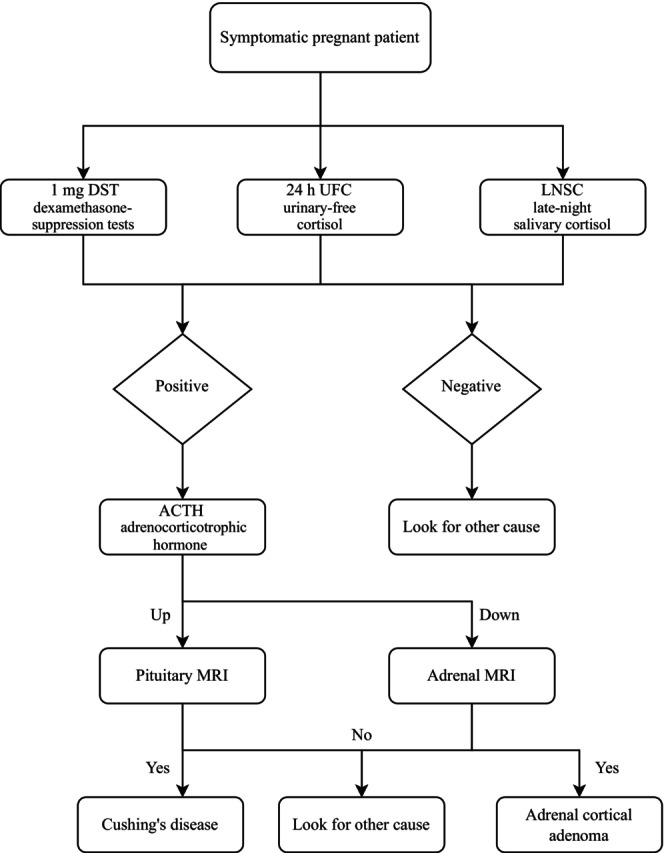
Assessment approach of CS in pregnancy.

Clarifying the causes of CS can be particularly challenging, especially during pregnancy. In nonpregnant individuals, ACTH is important for distinguishing between ACTH‐dependent CS and ACTH‐independent CS, and the former is characterised by an increase in ACTH levels. The increase in ACTH levels in pregnant women is closely linked to the placental synthesis and release of CRH and ACTH [5]. Therefore, the ability to inhibit serum ACTH levels is limited in primary adrenal CS during pregnancy. However, if the ACTH level is low, confirmation of adrenal CS may be present. In nonpregnant individuals, CRH, desmopressin and high‐dose dexamethasone suppression tests can be used as identification tests, but these do not apply during pregnancy. Imaging also plays a crucial role in identifying the cause of CS. When low or normal ACTH levels are observed alongside confirmed hypercortisolism, an adrenal MRI without contrast can be a valuable diagnostic step to identify adrenal tumours. In some cases, an adrenal ultrasound may also provide helpful information [27, 28] (Figure 3).

### 3.5 Management

Active CS can have severe consequences for both maternal and foetal health. A large review suggests that the maternal mortality rate for active CS during pregnancy is 2.0% [8]. Furthermore, CS during pregnancy can increase the risk of various complications, including miscarriage, preterm delivery, hypertension, gestational diabetes, heart failure and pulmonary oedema [29]. A definitive conclusion regarding the management of CS during pregnancy cannot be drawn due to the absence of uniform therapeutic consensus. Therefore, the treatment of CS in pregnant individuals requires personalised and customised approaches based on the specific cause of CS, the severity of hypercortisolism, the trimester of pregnancy and the possible therapy‐related complications [25]. Many reports highlight the clinical challenge of treating adrenal tumours during pregnancy. Surgical intervention is typically prioritised as the primary treatment approach [30]. However, medical treatment can be considered as a secondary therapeutic option in cases where surgery is contraindicated, unsuccessful or declined by the patient. Moreover, medical treatment may also be warranted for severe hypercortisolism associated with CS. Metyrapone, a drug commonly utilised for the treatment of CS, is an inhibitor of steroidogenesis and effectively hinders steroid production in the adrenal gland via CYPB11B1. The administration of this medication has been found to have no detrimental impact on foetal development. However, it is important to note that its usage is associated with an elevated risk of hypertension, hypokalaemia and preeclampsia [12]. In addition, the drug treatments encompass ketoconazole, mitotane, aminoglutethimide and mifepristone, and an antiglucocorticoid receptor antagonist that can ameliorate the signs and symptoms of Cushing's syndrome is not available in pregnancy. These medications are contraindicated in pregnancy due to potential adverse reactions, for instance, impacts on the developing foetus, leading to antiandrogenic, virilisation even miscarriage [31, 32].

Recent studies have shown that laparoscopic adrenalectomy may be the more effective treatment option and is associated with a higher neonatal survival rate [6, 8, 22, 33]. Unless there are indications such as maternal infection, psychosis, unstable vital signs and presence of comorbidities requiring urgent management, have surgical adrenalectomy [34]. There are two surgical approaches to performing adrenalectomy: laparoscopic transabdominal adrenalectomy (TA) and posterior retroperitoneoscopic approach (PRA). PRA offers potential benefits for pregnant patients due to no chance of injuring the uterus. It requires the gravid patient to be at a lateral decubitus position, which relieves uterine compression on the vena cava and results in enhanced oxygenation and improved systolic blood pressure [30]. However, the optimal timing for adrenalectomy remains uncertain due to the limited literature and a lack of consensus. Nevertheless, most obstetricians and surgeons generally agree that mid‐gestation is the most suitable period for performing laparoscopic adrenalectomy [33, 35]. Adrenalectomy has also been reported during late pregnancy. The risks of the procedure become more apparent as pregnancy progresses due to the increasing size of the uterus. This can make the operation more difficult, with potential risks including obstructed vision, restricted space, damage to surrounding organs and increased intra‐abdominal pressure. These factors can also affect uterine blood flow and potentially lead to acidosis from carbon dioxide absorption [36]. To ensure comprehensive perioperative management, it is essential to have a multidisciplinary team comprising surgeons, obstetricians, paediatricians, endocrinologists, cardiologists, anaesthesiologists and other relevant clinical specialists. This team is essential in preventing potential complications for both the mother and the infant [6]. Postoperative management is crucial and involves monitoring blood pressure, blood glucose, potassium levels and fluid balance.

As a result of hypercortisolism and the suppression of ACTH, patients commonly experience contralateral adrenal atrophy and tertiary hypoadrenalism after undergoing adrenalectomy. However, its nonspecific symptoms are easily overlooked, including nausea, vomiting, fatigue, and dizziness. In some severe cases, these symptoms can worsen and lead to shock, coma or even death. As a result, it is extremely important hormone replacement therapy after surgery and consistently monitors cortisol levels to track the recovery of the HPA axis. The administration of hydrocortisone as a glucocorticoid replacement therapy is recommended. This treatment aims to address the deficit in cortisol caused by corticotrope insufficiency. It is crucial to provide comprehensive education to the patient regarding adrenal insufficiency, ensuring that they have a thorough understanding of their condition and the necessary management strategies [9, 37].

The common complications, hypertension and diabetes, usually resolve or ameliorate after surgery or medication therapy of CS. However, it is usually necessary to take blood pressure medication during treatment [1]. Combination therapy with adrenergic blockers and calcium channel blockers may be useful to control blood pressure [38]. Most diuretics are typically not administered during pregnancy to manage blood pressure to avoid haemoconcentration and a decrease in effective circulation, especially thiazides, which may potentially worsen hypokalaemia. However, spironolactone may be used for the control of blood pressure, the amelioration of hypokalaemia and the contribution to antiandrogenic effects in women [39]. Some individuals have suggested utilising angiotensin‐converting enzyme inhibitors (ACEI) or angiotensin receptor blockers (ARB) as the initial treatment option due to their potential for cardiovascular protection, but it is prohibited in pregnancy [40].

CS in pregnancy is often associated with elevated blood glucose levels, which may be related to increased cortisol and the presence of insulin resistance [41]. Thus, the first step in enhancing glucose metabolism in individuals with endogenous CS is to regulate hypercortisolism [42]. Drugs that inhibit steroidogenesis, such as metyrapone, and medications that antagonise the glucocorticoid receptor, such as mifepristone, have a positive impact on glucose metabolism to enhance insulin sensitivity and control glucose, but mifepristone is prohibited in pregnancy. Meanwhile, availably controlled blood glucose needs antidiabetic treatment. Metformin is commonly prescribed as the first‐line therapy due to the dominant influence of hypercortisolism on insulin resistance. However, insulin therapy may be necessary instead of other drugs if the glucose level in blood is hazardous, especially considering safety during pregnancy [43].

## 4 Conclusion

CS in pregnancy caused by adrenal cortical adenoma is a rare condition that is easily misdiagnosed as hypertensive disorders of pregnancy (HDP) and presents substantial risks to the health of both the mother and the foetus, resulting in increased morbidity and mortality. Therefore, it is crucial to promptly recognise, diagnose and treat this condition. The optimum therapeutic strategy for this disease is currently lacking. The preferred approach for treatment is laparoscopic adrenalectomy during the second trimester. By implementing a multidisciplinary team consisting of urologists, obstetricians, paediatricians, endocrinologists, cardiologists and anaesthesiologists, CS in pregnant women can be effectively managed, leading to improved outcomes for both the mother and the foetus.

## Author Contributions

**Yan Wang:** Data curation (equal); formal analysis (equal); writing – original draft (equal); writing – review and editing (equal). **Yufen An:** Resources (equal). **Xiaomei Hou:** Supervision (equal). **Yanan Xu:** Validation (equal). **Zhen Li:** Software (equal). **Xin Liu:** Supervision (equal). **Fumin Zheng:** Methodology (equal). **Mingze Sun:** Investigation (equal). **Rendong Han:** Investigation (equal). **Caixia Lu:** Writing – original draft (equal). **Jing Li:** Writing – review and editing (equal). **Jun Zhou:** Writing – review and editing (lead).

## Ethics Statement

The studies involving human participants were reviewed and approved by the Ethics Committee of the Affiliated Hospital of Qingdao University.

## Consent

The patients provided their written informed consent to participate in this study. Written informed consent was also obtained from the individuals for the publication of any identifiable images or data included in this article.

## Conflicts of Interest

The authors declare that the research was conducted in the absence of any commercial or financial relationships that could be construed as a potential conflict of interest.

## Data Availability

The original contributions presented in the study are included in the article/supplementary material. Further inquiries can be directed to the corresponding author.
